# Follicular CD4 T Helper Cells As a Major HIV Reservoir Compartment: A Molecular Perspective

**DOI:** 10.3389/fimmu.2018.00895

**Published:** 2018-06-18

**Authors:** Malika Aid, Frank P. Dupuy, Eirini Moysi, Susan Moir, Elias K. Haddad, Jacob D. Estes, Rafick Pierre Sekaly, Constantinos Petrovas, Susan Pereira Ribeiro

**Affiliations:** ^1^Beth Israel Deaconess Medical Center, Center for Virology and Vaccine Research, Harvard Medical School, Boston, MA, United States; ^2^Centre hospitalier de l’Université de Montréal, Montreal, QC, United States; ^3^Tissue Analysis Core, Immunology Laboratory, Vaccine Research Center, NIH, Bethesda, MD, United States; ^4^Laboratory of Immunoregulation, National Institute of Allergy and Infectious Diseases (NIAID), Bethesda, MD, United States; ^5^Division of Infectious Diseases & HIV Medicine, Department of Medicine, Drexel University College of Medicine, Philadelphia, PA, United States; ^6^Oregon National Primate Research Center, Vaccine and Gene Therapy Institute, Oregon Health & Science University, Beaverton, OR, United States; ^7^Pathology Department, Case Western Reserve University, Cleveland, OH, United States

**Keywords:** HIV, lymph nodes, TFH cell, cure, gene expression

## Abstract

Effective antiretroviral therapy (ART) has prevented the progression to AIDS and reduced HIV-related morbidities and mortality for the majority of infected individuals. However, a lifelong administration of ART is necessary, placing an inordinate burden on individuals and public health systems. Therefore, discovering therapeutic regimens able to eradicate or functionally cure HIV infection is of great importance. ART interruption leads to viral rebound highlighting the establishment and maintenance of a latent viral reservoir compartment even under long-term treatment. Follicular helper CD4 T cells (TFH) have been reported as a major cell compartment contributing to viral persistence, consequent to their susceptibility to infection and ability to release replication-competent new virions. Here, we discuss the molecular profiles and potential mechanisms that support the role of TFH cells as one of the major HIV reservoirs.

## Introduction

Antiretroviral therapy (ART) has impacted on the quality of life of HIV-infected subjects. However, the persistence of a long-lasting viral reservoir, where fewer than 10% of the infected cells harbor replication-competent provirus (1–4), poses a major obstacle for viral eradication. Recently, TFH cells have been reported as a potential sanctuary for HIV/SIV replication and an important compartment for viral persistence (5, 6), making them an important target for cure therapies.

## Germinal Center (GC) and Circulating (c) TFH Cells

**Figure 1:**
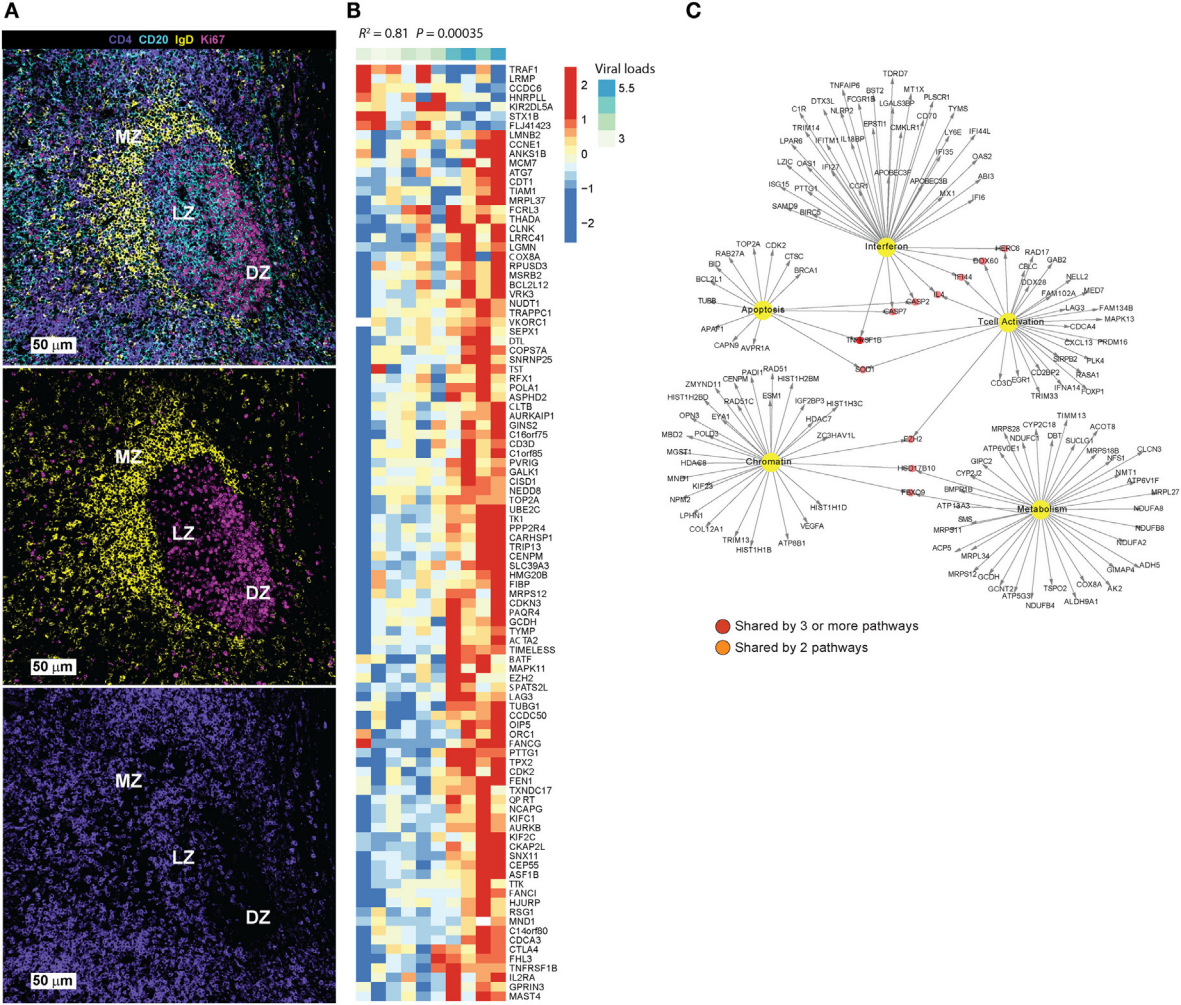
**(A)** Main areas of a tonsillar follicle defined by IgD (yellow), CD20 (cyan), and Ki67 (magenta) are shown: marginal zone (IgD^hl^CD20^dim^), germinal center light zone (IgD^neg^sCD20^hi^Ki67^hi/lo^) and dark zone (IgD^neg^PCD20^dim^Ki67^hi^). The distribution of CD4 T cells (purple) is shown in the lower image. **(B)** Differential gene expression analysis was performed using Limma model by comparing sorted TFH cells (CXCRS^high^) vs. Non-TFH (CXCR5^low^) from eight HIV- donors in LNs. A corrected *p*-value cutoff of 0.05 (BH method) was used to select significant upregulated genes (731 genes) in CXCR5^high^ vs. CXCR5^low^ TFH cells. Linear regression model to correlate these genes to viral load in a cohort of HIV viremic subjects (10 viremic subjects) was performed in R. Heatmap shows the log_2_ normalized expression of each gene transformed to *z*-score where the average expression of each gene was subtracted and divided by its SD across samples. Genes that correlated significantly to viral load (*p*-value <0.05) were shown on the heatmap. **(C)** Pathways enrichment analysis using genes in panel **(B)** and a compiled set of pathways from MsigDB C2 collection (http://software.broadinstitute.org/gsea/msigdb/collections.jsp#C2) and pathways from Chaussabel et al. (16) was performed. A FDR cutoff of 0.05 was used to selected pathways significantly correlated to viral loads. Interferon, metabolism, T cell activation and differentiation, apoptosis, and chromatin regulators were enriched. Cytoscape was used to infer gene-interacting networks of the leading genes of these pathways. Yellow nodes represent pathways name, red nodes represent genes shared by more than three pathways, orange nodes represent genes shared by two pathways, and white nodes represent genes specific to each pathway.

TFH cells localize specifically in B cell follicles (BCF) in secondary lymphoid organs (5, 6) and express a CCR7^lo^ CXCR5^hi^PD-1^hi^ICOS^hi^ BTLA^hi^CD69^hi^SAP^hi^ phenotype (7). Through surface receptors and soluble factors like IL-21 and IL-4 (8), TFH cells provide help for the survival, activation, and maturation of GC B cells. A complex network of transcription factors (TFs), including BCL-6, interferon regulatory factor (IRF4), c-Maf, and BATF, promote TFH differentiation while inhibiting alternate CD4 T cell differentiation pathways (9). The downregulation of CCR7 and upregulation of CXCR5 licenses the migration of activated T cells to BCFs of secondary lymphoid organs and promotes their interaction with B cells, further upregulating the expression of Bcl-6 and leading to the establishment of effector and memory TFH cell programs (10). The mutual regulation of TFH and B cells (11), through receptor–ligand interactions like ICOS/ICOSL, PD-1/PD-L1, and CD40/CD40L (11) and soluble mediators like IL-21 and IL-4 (12), is critical for the formation and maintenance of GC structure and provision of critical help for the development of antigen-specific B cell responses (13–15). TFH cells can populate different areas of the follicle, including the marginal zone that surrounds the GC and the “light” zone (Figure 1A).

The fate of memory TFH cells after a resolved infection, immunization, or response to foreign antigen is not known. Several studies have focused on the characterization of cTFH cells (17–20) and their ability to promote differentiation and/or class switching of autologous B cells (19). However, their lineage commitment and relationship to GC TFH cells is not well understood. Given the limited accessibility to lymph nodes during clinical trials, the identification and validation of blood biomarkers (21) that could provide robust estimation of GC reactivity is of great interest. In this context, the development of single cell sequencing, allowing for the TCR clonotypic analysis and global transcriptional profiling, would be instrumental for the understanding of the lineage relationship between cTFH and follicular TFH cells.

## Transcriptional Regulation of TFH Cell Differentiation

A complex network of signaling molecules and TFs has been described for the development and maintenance of TFH cells (9). In humans, the generation of TFH is promoted by the synergistic function of Bcl-6 and c-MAF (22). Several TFs can promote TFH differentiation in a Bcl-6-dependent (i.e., LEF1 and TCF1) (23) or -independent (i.e., ASCL2) (24) manner. On the other hand, Blimp1 represents a major TF that suppresses TFH differentiation, by modulating the expression of BCL-6 (25). KLF2 restricts the *in vivo* development of TFH, an activity mediated by the induction of S1PR1 and Blimp1 (26). How the course of HIV/SIV infection modulates this complex network of TFs is not well understood. To this end, longitudinal NHP studies will be highly informative (27).

Members of STAT family play a central role in TFH differentiation upon the engagement of receptors for γ-C cytokines which are required for TFH survival and differentiation. The cytokines IL-6 and IL-21, both positive regulators of TFH differentiation, induce BCL-6 expression through STAT-3 activation (28), while IL-27 acts likely *via* its indirect impact on IL-21 production (29). IRF4, expression of which is dependent on TCR signaling strength (30, 31), globally cooperates with STAT-3 (9) as a complex to regulate IL-21-mediated gene expression. In contrast to STAT-3, STAT-5 has a negative impact on TFH development at least by suppressing the expression of TFs like c-Maf, BCL-6, and Batf (25). IL-2 inhibits TFH differentiation by activating STAT-5 which prevents the binding of STAT-3 to the Bcl-6 promoter. Alternatively, STAT-5 deficiency greatly enhances TFH gene expression *in vitro*, in part associated to dampened Blimp1 expression (32). These observations highlight the inhibitory crosstalk that takes place between STAT-5/Blimp1 and the IL-6/IL21/STAT-3/BCL-6 pathways in TFH development (10). In addition to STAT-3 and STAT-5, several studies have indicated that STAT-1 and STAT-4 are also involved in TFH differentiation. IL-6 and IFNg induced STAT-1 was shown be required for BCL-6 induction of early TFH differentiation *in vivo* (33, 34). Additionally, IL-12-mediated STAT-4 activation can induce expression of IL-21 and BCL-6 to generate cells with features of both TFH and Th1 cells (35). Altogether, these findings indicate that the interactions among TFs that determine the fate of specialized CD4^+^ T-cell lineages are complex, giving them flexibility and potential to respond to environmental conditions by altering the expression of critical specific TFs as needed.

## GC Dynamics in HIV/SIV Infection

The GC dynamics in HIV infection is a subject of intense research. The susceptibility of TFH cells to infection (36), the local inflammatory microenvironment (37, 38) and potential sequestration of innate and pro-inflammatory cells (39, 40), as well as their close proximity to Follicular Dendritic Cells (FDCs) that harbor infectious virus for long periods of time (41–43) represent biological factors that could contribute to TFH cell dynamics during the course of HIV/SIV infection. Acute SIV infection is characterized by modest increases in the relative frequency of TFH cells (36, 44, 45) while chronic viremia has a dramatic effect on extrafollicular and follicular architecture and TFH dynamics affecting the development of HIV/SIV specific antibody responses (46). Available viral antigen, possible preferential deletion of Env-specific TFH CD4 T cells, loss of stromal cells like fibroblastic reticular cells (47) that directly affects the dynamics of T cells (47) and their trafficking within lymph node areas (48) and altered tissue architecture due to progressive deposition of fibrotic collagen (49), a major determinant of altered LN architecture (47, 49, 50), could contribute to altered GC T-B cell interactions with direct implications for the development of broadly neutralizing antibodies. In fact, circulating GC-related factors like CXCL-13 have been proposed for monitoring the development of such antibodies (21, 51). In the advanced phase of disease (AIDS), significantly lower frequencies of TFH cells were found indicating accelerated loss of TFH cells under these conditions (52) when compared to other CD4 subsets. TFH cells express unusually high levels of the co-inhibitory receptor PD-1 further sensitizing them to “pre-apoptotic” signals (53) upon interaction with locally expressed PD-1 ligands during chronic infection (54). Whether the loss of TFH cells is due to their accelerated “exhaustion” associated with AIDS, an increased operation of pre-apoptotic pathways, or a result of an advanced loss of structure and vital signals (50) is not known and needs further investigation. The delineation of local pro- and anti-inflammatory networks will further inform on the cellular and molecular mechanisms governing the dynamics of TFH cells in chronic infection and might lead to novel strategies for virus elimination by manipulating such pathways. Thus, although early ART rapidly controls HIV/SIV replication, it only partially reduces lymphoid and systemic markers of cellular activation, resulting in increased TFH frequencies and persistent hyperplastic BCFs, which may contribute to the seeding and magnitude of viral reservoirs within these lymphoid tissue compartments (55).

## TFH Cells: A Preferential HIV/SIV Reservoir

TFH cells have been demonstrated to be both productively (i.e., vRNA^+^) and latently (i.e., vDNA) infected at higher frequencies (5, 56) than non-TFH cells. TFH cells are in the vicinity of cells harboring virus-immune complexes like FDCs and B cells (57–59) that serve as a significant reservoir and are able to effectively infect CD4^+^ T cells *in vitro* (41, 60, 61). Their unique localization in combination with their activation status could contribute to the higher infection levels of TFH cells *in vivo*. *In vitro* studies have further supported the preferential infection (5) and production of infectious virus from TFH compared to non-TFH cells (5, 6, 43). HIV RNA was found in TFH cells even after ART initiation, although those levels were lower in long-term ART treated donors (6). Similar results were found during chronic SIV infection (44, 62). Recently, follicular regulatory T (TFR) cells, shown by gene profiling as not originated from the same lineage as TFH cells (63, 64), share phenotypic characteristics with TFHs and are even more permissive to HIV-1 infection both *ex vivo* and *in vivo*. This can be in part mediated by the higher surface expression of CCR5 and CD4 (65) and their higher activation and *in vivo* cycling status, judged by the expression of Ki67 (65). Given the shared surface characteristics with TFH cells, TFR cells might contribute to data ascribed as specific for TFHs. However, what impacts the preferential infection of TFH and TFRs cells *in vivo* is not well understood and warrants further investigation. Generation of viral targets due to TFH cell accumulation in chronic infection (64), their unique cellular profile, the low penetrance of ART in the tissues (66), the relative exclusion of virus-specific CD8 T cells from the BCFs and particularly the GCs (67, 68), as well as the expression of local cytokines like IL-10 (69), TGFb (47) that interfere with the activation of CD4 T cells or chemokines involved in HIV infection like CXCL9 (70) could represent biological factors contributing to the preferential establishment of virus persistence in TFH cells (70). Further delineation of the cellular and molecular mechanisms underlying TFH dynamics will inform the design of novel therapies for viral elimination. However, novel therapeutics targeting the virus reactivation in TFH should be carefully evaluated given the unique biology and localization of TFH.

## Molecular Pathways Promoting TFH Susceptibility to HIV Infection and Reservoir Establishment

In an attempt to understand better the preferential infection of TFH cells, we performed microarray analysis on TFH (CD4^+^CXCR5^high^) and non-TFH (CD4^+^CXCR5^low^) T cells sorted from lymph nodes from healthy subjects (not published data). Differential gene expression analysis of TFH (CD4^+^CXCR5^high^) compared to non-TFH (CD4^+^CXCR5^low^) revealed upregulation of major TFH markers (CXCR5, PDCD1, MAF, BCL-6, LAG3, IRF4) (data not shown).

In order to identify TFH genes and pathways associated with HIV replication and/or reservoir establishment, we performed a linear regression analysis using a compiled set of TFH signatures (see Figures 1B,C legend for more details) with integrated DNA (IntDNA—latent reservoir), TILDA [inducible HIV reservoir Ref. (71)], and plasma viral load (VL—active replicating virus) measurements from a cohort of HIV viremic subjects. We found a significant and positive correlation of several TFH specific signatures to IntDNA, TILDA, and VL (data not shown). This regression analysis was performed in viremic subjects, allowing us to correlate specific pathways of gene expression to active viral replication. The TFH genes associated to high viral load are shown (Figure 1B). Pathways enrichment analysis using these genes was performed using MsigDB C2 collection and pathways from Chaussabel et al. (16). We observed an enrichment in genes related to modulation of T cell activation, co-stimulation and MHC protein binding (Lag3, CD3D, NELL2, FAM102A), apoptosis (CASP2, CASP7, APAF1, BID, associated to positive regulation of programmed cell death, intrinsic apoptotic signaling), metabolism (NUDFB8, NDUFA2, NDYFB8, NDUFC1, all associated with NADH dehydrogenase activity and ATP synthesis coupled electron transport), chromatin organization (RAD51, RAD51C, POLD3, NPM2, associated with DNA polymerase activity and DNA repair), and interferon genes (CASP7, IFI44, DDX60, OAS1, OAS2, ISG15, associated with response to virus, regulation of viral genome replication, IFNg mediated signaling) (Figure 1C). The presence of genes related to T cell activation would support the hypothesis that TFH cells are more prone to infection; this is confirmed by the expression in TFH of genes related to DNA polymerase and DNA repair from the chromatin module. Epigenetic modifications as shown by the expression of permissive (Ex. H3K4me3) or repressive (Ex. H2K27me3) chromatin modifications, have been reported as modulating T cell fate (29) and can have an impact on HIV integration and expression.

**Figure 2:**
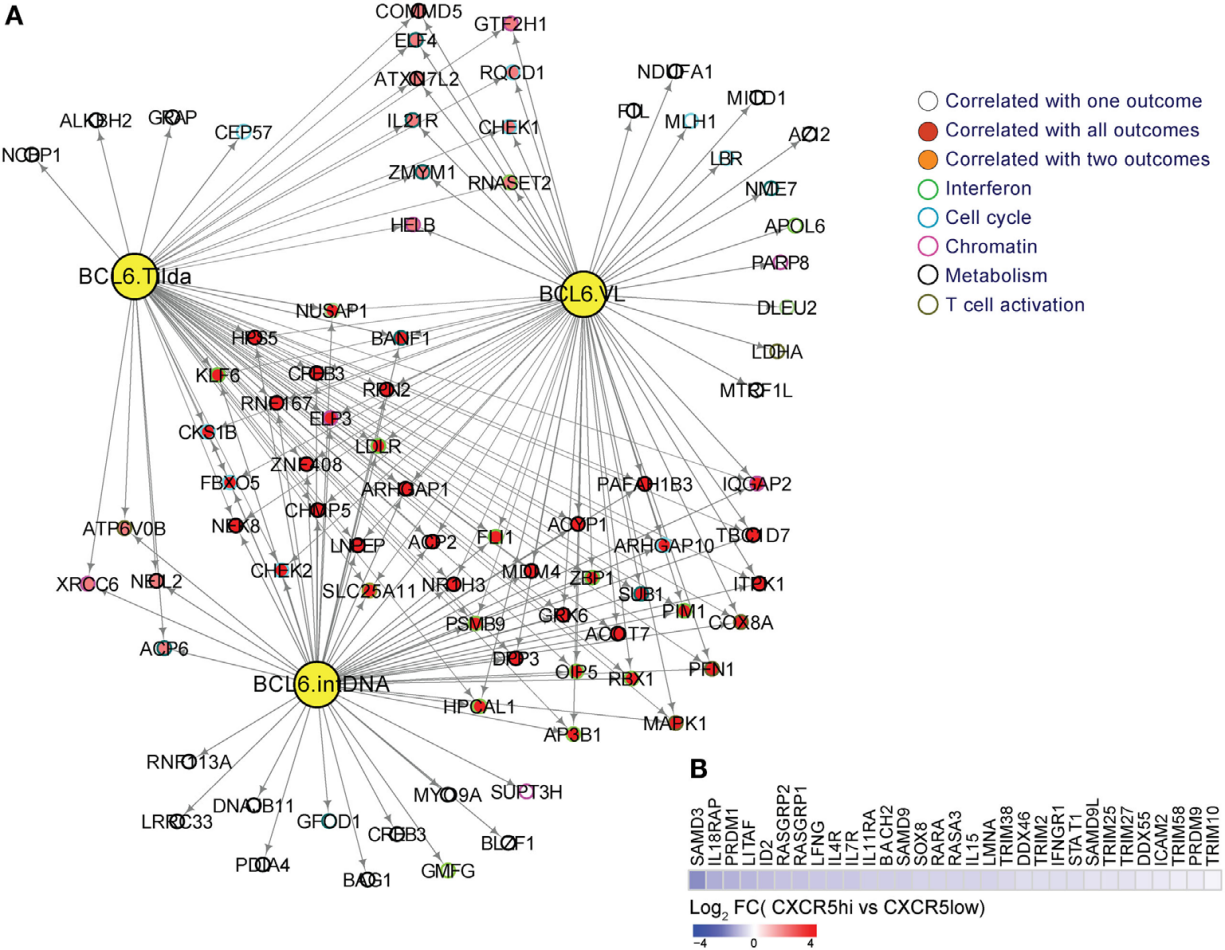
**(A)** BCL-6 target genes are enriched in genes from interferon, metabolism, T cell activation and differentiation, cell cycle, and chromatin regulators pathways. A linear regression model to correlate BCL-6 targets to viral load, integrated DNA, and inducible RNA (TILDA) from a cohort of viremic subjects was performed using R. BCL-6 targets correlated significantly to viral load, integrated DNA and TILDA (*p*-value <0.05) were used to infer a gene-interacting network using Cytoscape. Yellow nodes represent BCL-6 targets correlated respectively to intDNA, TILDA, and viral loads. Red nodes represent genes correlated to viral load, integrated DNA, and TILDA, orange nodes represent genes correlated to two pathways and white nodes represent genes specific to each outcome. Circles color depict the different molecular functions enriched in these genes; **(B)** downregulation of restriction factors and genes involved in interferon type 1 signaling in CXCRS^high^ vs. CXCR5^low^ cells from TFH cells from HIV^−^ donors in LNs. Heatmap representing the gene log_2_-fold change expression ranging from blue ( down regulation) to red ( upregulation).

The transcriptional suppressor BCL-6 has been shown to suppress the expression of antiviral genes in TFH cells (61, 72). Indeed, several genes are under direct or indirect control of BCL-6 and are positively correlated to the maintenance of HIV reservoir (IntDNA) and to the other viral outcomes (TILDA and VL) (Figure 2A). Conversely, silencing viral gene expression allows the survival of infected cells *via* two mechanisms: (i) the diminished viral gene expression downregulates viral production, which in turn prevents the virus-induced cytopathic effect and (ii) the reduced antigen presentation on MHC-I prevents recognition by CTLs or natural killer cells and, therefore, prevents cell-mediated cytotoxic killing (61). The low frequency of HIV-specific CD8 T cells in GC (67), their impaired cytotoxic capacity compared to their blood counterparts (70) or even their inability to sense the infected cells could represent cellular mechanisms for this outcome. Future studies are needed to understand the mechanism of viral gene suppression and to determine if inducing the expression of antiviral genes could lead to apoptosis of the infected cells and, therefore, the reduction of viral reservoir. Alternative, recent reports have indicated (73, 74) that combinatorial approaches aiming at virus reactivation and infected cell elimination by immunotherapies like bispecific antibodies could represent promising strategies in the context of HIV cure.

## Interferon Family of Genes and its Expression Profiles in TFH Cells from Viremic Subjects

Analysis of the type I IFN component in human TFH cells has shown a decreased expression of genes related to restriction factors (Figure 2B). This is corroborated by previous findings where microarray data sets (GEO #GSE50391) of tonsil TFH cells revealed that human TFH cells exhibit diminished expression of several anti-HIV restriction factors, including MX2, IFITMs, SAMHD1, and SLFN11, which are the IFN-stimulated genes (ISGs) shown to inhibit HIV infection/replication (75, 76). Additionally, BCL-6 has been reported to inhibit IRF7, an important antiviral TF (15, 77), and might thus contribute to the lack of intrinsic antiviral immunity in TFH cells. This fact can lead to sustained virus infection, replication, and integration, which combined with lower immune pressure could lead to the establishment of intact DNA in this T cell compartment. Indeed, diminished constitutive expression of ISGs including the antiviral resistance factor MX dynamin-like GTPase 2 (MX2) and IFN-induced transmembrane 3 (IFITM3) in TFH compared with non-TFH cells might contribute to their higher susceptibility to HIV infection as previously reported. The lower antiviral resistance of TFH is consistent with a profile of higher susceptibility to retroviral infections (15). In addition, the lack of intracellular host restriction factors, such as SAMHD1, was also reported to enhance infection with high degree of viral replication (78). Additionally, BCL-6 binds to ISG loci and inhibits the expression of MX2 and IFITM3 in TFH cells. The ability of BCL-6 to control these pathways impacts directly on the TFH susceptibility to HIV infection. Amet et al. (15) demonstrated that inhibition of the BCL-6, BR-C, ttk, and bab (BTB) domain function increased the expression of ISGs and suppressed HIV infection and replication in TFH cells, revealing a regulatory role of BCL-6 in inhibiting antiviral resistance factors, thereby promoting TFH susceptibility to viral infections (15). These suggests that the modulation of BCL-6 function in TFH cells could be a potential strategy to enhance TFH cell resistance to retroviral infections and potentially decrease cellular reservoirs during HIV infection (15).

## Conclusion

HIV infection leads to hyperplastic BCFs and increased TFH cell frequencies. TFH cells have been reported to represent an important HIV reservoir compartment harboring intact and infective proviruses. BCL-6 is the master regulator of TFH cell differentiation and plays a role in the modulation of a series of other TFs and their downstream targets. Among those genes are the ones related to IFNs, cell cycle, chromatin modifiers, metabolism, and T cell activation. All these genes play a role in the heightened susceptibility of TFH cells to infection as well as the integration of intact provirus. Further understanding of molecular pathways and genes involved in the establishment of viral infection in TFH cells could represent a strategy for efficient depletion of the HIV reservoir in this T cell compartment.

## Author Contributions

MA: data analyses and interpretation, FD and EM: data generation, SM: data analysis and writing, EH and JE: data generation and writing, RS and CP: writing, SR: review outline, guidance on data analysis, and writing.

## Conflict of Interest Statement

The authors declare that the research was conducted in the absence of any commercial or financial relationships that could be construed as a potential conflict of interest.
